# Supplementary material for: Migrating birds optimization-based feature selection for text classification
Source: PeerJ Comput Sci. 2024 Aug 30;10:e2263. doi: 10.7717/peerj-cs.2263 (PMC11639163; doi:10.7717/peerj-cs.2263)
Supplement: Supplemental Information 1 [file peerj-cs-10-2263-s001.zip › README COPYRIGHT.pdf]

This project is part of an unpublished work and is the intellectual property of Cem Kaya. Unauthorized reproduction, distribution, or disclosure of this code or its contents is strictly prohibited. Any use of this code without express written permission from Cem Kaya is prohibited and may be subject to legal action.
